# Supplementary material for: The Influence of Adipocyte Secretome on Selected Metabolic Fingerprints of Breast Cancer Cell Lines Representing the Four Major Breast Cancer Subtypes
Source: Cells. 2023 Aug 22;12(17):2123. doi: 10.3390/cells12172123 (PMC10486438; doi:10.3390/cells12172123)
Supplement: Supplementary file 1 [file cells-12-02123-s001.zip › cells-2505876-supplementary.pdf]

**Table S1 – Nutrient composition of cell medium of control (DMEM) and treatment (secretome).**

| Metabolite | Control (DMEM) | Treatment (secretome) |
|---|---|---|
| Glucose (mM) | 20 | 15 |
| Pyruvate (mM) | 0.3 | 0.03 |
| Lactate (mM) | 0 | 1.2 |
| Acetate (mM) | 0.09 | 0.2 |
| Alanine (mM) | 0 | 0.6 |
| Glutamine (mM) | 6 | 1.6 |
| Glutamate (mM) | 0 | 0 |
| Isoleucine (mM) | 0.7 | 0.4 |
| Valine (mM) | 0.7 | 0.4 |
| Triglycerides (mg/dL) | 1 | 53 |
| Cholesterol (mg/dL) | 21 | 24 |

**Table S2 – Expression of beta actin on mRNA level from the performed RT-PCR.**

| | CT PCR1 | CT PCR2 | CT PCR3 | Final CT | 2^(-CT) |
|---|---|---|---|---|---|
| MCF7 C1 | 18,3377 | 18,77367 | 18,41137 | 18,50758 | 2,68327E-06 |
| MCF7 C2 | 18,3295 | 19,17043 | 18,61358 | 18,7045 | 2,34091E-06 |
| MCF7 C3 | 18,38988 | 19,68531 | 18,46581 | 18,847 | 2,12074E-06 |
| MCF7 C4 | 18,58482 | 19,69935 | 18,74795 | 19,0107 | 1,89325E-06 |
| MCF7 C5 | 18,85521 | 19,73949 | 19,01118 | 19,20196 | 1,65819E-06 |
| MDA 231 C1 | 18,43274 | 19,1138 | 18,64384 | 18,73013 | 2,29969E-06 |
| MDA 231 C2 | 18,41857 | 18,8613 | 18,42292 | 18,56759 | 2,57393E-06 |
| MDA 231 C3 | 18,5863 | 19,12144 | 18,63262 | 18,78012 | 2,22136E-06 |
| MDA 231 C4 | 17,51715 | 18,99463 | 18,2226 | 18,2448 | 3,21936E-06 |
| MDA 231 C5 | 18,24021 | 19,19319 | 18,39148 | 18,60829 | 2,50234E-06 |
| BT474 C2 | 13,68 | 13,235 | 13,205 | 13,37333 | 9,42379E-05 |
| BT474 C3 | 13,58 | 13,31 | 13,25 | 13,38 | 9,38034E-05 |
| BT474 C4 | 14,14 | 13,77 | 13,65 | 13,85333 | 6,75665E-05 |
| BT474 C5 | 13,56 | 13,33 | 13,37 | 13,42 | 9,12384E-05 |
| SKBR3 C1 | 17,98981 | 18,4534 | | 18,22161 | 3,27151E-06 |
| SKBR3 C2 | 15,33128 | 15,44609 | 15,89798 | 15,55845 | 2,07224E-05 |
| SKBR3 C3 | 18,10436 | 17,99487 | 17,98436 | 18,02786 | 3,74173E-06 |
| SKBR3 C4 | 16,38149 | 16,77188 | 16,65473 | 16,6027 | 1,00482E-05 |
| SKBR3 C5 | 15,78928 | 15,90693 | 15,97893 | 15,89171 | 1,64482E-05 |
| MCF7 T1 | 17,32354 | 19,47494 | 17,88758 | 18,22869 | 3,25551E-06 |
| MCF7 T2 | 18,04393 | 18,9345 | 18,11221 | 18,36355 | 2,96498E-06 |
| MCF7 T3 | 17,96866 | 19,3034 | 17,58455 | 18,28554 | 3,12972E-06 |
| MCFT T4 | 18,14206 | 19,25178 | 17,62466 | 18,3395 | 3,01481E-06 |
| MCFT T5 | 17,68686 | 19,05613 | 17,23485 | 17,99261 | 3,83428E-06 |
| MDA 231 T1 | 17,34028 | 19,38313 | 18,26016 | 18,32785 | 3,03925E-06 |
| MDA 231 T2 | 18,36129 | 18,75279 | 18,47408 | 18,52939 | 2,64301E-06 |
| MDA 231 T3 | 17,95972 | 18,79696 | 18,27936 | 18,34535 | 3,00262E-06 |
| MDA 231 T4 | 17,76845 | 18,79349 | 18,21941 | 18,26045 | 3,18462E-06 |
| MDA 231 T5 | 17,08262 | 18,42048 | 17,96621 | 17,82311 | 4,31231E-06 |
| BT474 T2 | 13,69 | 13,56 | 13,45 | 13,56667 | 8,24188E-05 |
| BT474 T3 | 14,2 | 14,005 | 13,86 | 14,02167 | 6,01254E-05 |
| BT474 T4 | 13,285 | 13,2 | 13,26 | 13,24833 | 0,000102767 |
| BT474 T5 | 14,39 | 14,085 | 13,99 | 14,155 | 5,48176E-05 |
| SKBR3 T1 | 17,25593 | 17,45463 | 17,36785 | 17,35947 | 5,94674E-06 |
| SKBR3 T2 | 16,27944 | 16,80253 | 16,74227 | 16,60808 | 1,00108E-05 |
| SKBR3 T3 | 17,21542 | 17,43576 | 17,28724 | 17,31281 | 6,14221E-06 |
| SKBR3 T4 | 17,68618 | 17,21191 | 17,1729 | 17,357 | 5,95694E-06 |
| SKBR3 T5 | 16,33717 | 16,3909 | 16,52253 | 16,41687 | 1,14296E-05 |

NOTE: Average of the assays was taken in account to calculate 2^(-CT).
